# Evidence-Based Treatment Options in Recurrent and/or Metastatic Squamous Cell Carcinoma of the Head and Neck

**DOI:** 10.3389/fonc.2017.00072

**Published:** 2017-05-09

**Authors:** Athanassios Argiris, Kevin J. Harrington, Makoto Tahara, Jeltje Schulten, Pauline Chomette, Ana Ferreira Castro, Lisa Licitra

**Affiliations:** ^1^Hygeia Hospital, Athens, Greece; ^2^Thomas Jefferson University, Philadelphia, PA, USA; ^3^Division of Radiotherapy and Imaging, The Institute of Cancer Research, London, UK; ^4^Department of Head and Neck Medical Oncology, National Cancer Center Hospital East, Tokyo, Japan; ^5^Merck KGaA, Darmstadt, Germany; ^6^Centro Hospitalar do Porto, Porto, Portugal; ^7^Department of Head and Neck Cancer Medical Oncology, Fondazione IRCCS Istituto Nazionale Tumori, University of Milan, Milan, Italy

**Keywords:** cetuximab, squamous cell carcinoma of the head and neck, immune checkpoint inhibitor, EXTREME, platinum-refractory, recurrent and/or metastatic, programmed cell death protein 1, programmed cell death ligand 1

## Abstract

The major development of the past decade in the first-line treatment of recurrent and/or metastatic squamous cell carcinoma of the head and neck (R/M SCCHN) was the introduction of cetuximab in combination with platinum plus 5-fluorouracil chemotherapy (CT), followed by maintenance cetuximab (the “EXTREME” regimen). This regimen is supported by a phase 3 randomized trial and subsequent observational studies, and it confers well-documented survival benefits, with median survival ranging between approximately 10 and 14 months, overall response rates between 36 and 44%, and disease control rates of over 80%. Furthermore, as indicated by patient-reported outcome measures, the addition of cetuximab to platinum-based CT leads to a significant reduction in pain and problems with social eating and speech. Conversely, until very recently, there has been a lack of evidence-based second-line treatment options, and the therapies that have been available have shown low response rates and poor survival outcomes. Presently, a promising new treatment option in R/M SCCHN has emerged: immune checkpoint inhibitors (ICIs), which have demonstrated favorable results in second-line clinical trials. Nivolumab and pembrolizumab are the first two ICIs that were approved by the US Food and Drug Administration. We note that the trials that showed benefit with ICIs included not only patients who previously received ≥1 platinum-based regimens for R/M SCCHN but also patients who experienced recurrence within 6 months after combined modality therapy with a platinum agent for locally advanced disease. In this review, we outline the available clinical and observational evidence for the EXTREME regimen and the initial results from clinical trials for ICIs in patients with R/M SCCHN. We propose that these treatment options can be integrated into a new continuum of care paradigm, with first-line EXTREME regimen followed by second-line ICIs. A number of ongoing clinical trials are comparing regimens with ICIs, alone and in combination with other ICIs or CT, with the EXTREME regimen for first-line treatment of R/M SCCHN. As we eagerly await the results of these trials, the EXTREME regimen remains the standard of care for the first-line treatment of R/M SCCHN.

## Introduction

Head and neck cancer accounts for over 500,000 new cases and nearly 300,000 deaths annually worldwide as of 2012 (1, 2). Treatment options for patients with this disease vary according to the disease setting as well as other clinical characteristics. Patients with localized squamous cell carcinoma of the head and neck (SCCHN) (American Joint Committee on Cancer stages I-IVB) are treated with potentially curative therapy using ≥1 treatment modalities [surgery, radiation therapy, chemotherapy (CT), and biologic therapy]. However, many patients develop recurrent disease; the recurrence rate in early-stage SCCHN is ≈10–20% (3), whereas the recurrence rate in locally advanced (LA) SCCHN is ≈50% with a predominance of locoregional failure (4–6). Patients with recurrent or metastatic (R/M) SCCHN have a poor prognosis with median overall survival (OS) of under 1 year (7). This population includes patients whose disease recurred locally or who developed distant metastasis after initial treatment for localized disease and the rare patients with distant metastasis at first presentation. A small percentage of patients with localized recurrence can be treated with curative intent, but the vast majority receive palliative treatment with systemic therapy. In the first-line treatment of R/M SCCHN, combination therapy with cetuximab plus cisplatin/carboplatin plus 5-fluorouracil (5-FU) followed by maintenance cetuximab (the “EXTREME” regimen) has shown the best results so far in terms of overall response rate (ORR), progression-free survival (PFS), and OS (8, 9). A variation on this regimen allows for the substitution of 5-FU for a taxane (e.g., docetaxel or paclitaxel) (10, 11). In clinical practice, other combinations, such as a taxane or cisplatin plus cetuximab, are also sometimes used as first-line treatment for R/M SCCHN when patients are not fit enough for the EXTREME regimen, even though these are not evidence-based approaches.

Patients who progress on—or are ineligible for—the EXTREME regimen and other cetuximab-based first-line treatments have a dearth of efficacious therapeutic options. ORRs to commonly used therapies (including methotrexate, docetaxel, paclitaxel, and cetuximab as monotherapies) drop off to well under 20% and median survival in phase 3 trials has been reproducibly reported at ≈5–6 months (7, 12–16). This grim outlook for second-line treatment is being reshaped by the introduction of immune checkpoint inhibitors (ICIs); results of recent trials will be reviewed here.

As more treatment options become available, it is reasonable to propose that the outcomes for patients with R/M SCCHN could be optimized with the appropriate succession of treatment regimens. Maximizing the number of therapy lines and optimizing the order in which therapies are administered has been one of the most powerful tools for delivering maximum benefit to patients (17, 18). Therefore, it is important to integrate as many lines of potentially efficacious therapy as possible into the treatment paradigm to generate a maximally effective and tolerable multi-line continuum of care.

**Figure 1:**
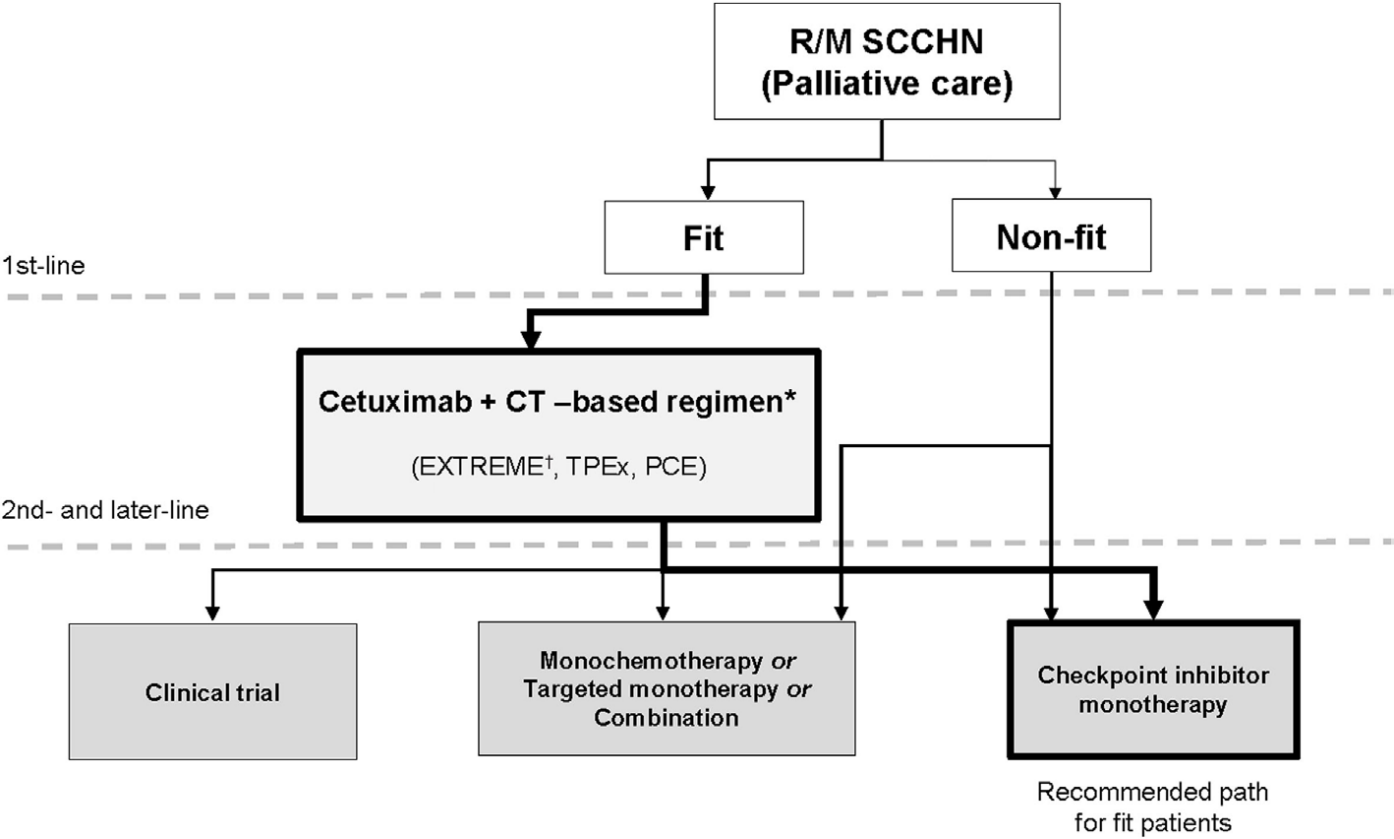
**New continuum of care for R/M SCCHN**. New drugs are under investigation in SCCHN and will change the treatment landscape for R/M disease. There are now multiple lines of treatment that constitute a continuum of care. The objective of this paper is to define the position of these new drugs in the current treatment landscape. The algorithm for unfit patients’ needs to be further established in prospective trials. CT, chemotherapy; EXTREME, cetuximab plus cisplatin/carboplatin plus 5-fluorouracil followed by maintenance cetuximab; PCE, paclitaxel, carboplatin, and cetuximab, followed by cetuximab maintenance until progressive disease or toxicity; R/M, recurrent and/or metastatic; SCCHN, squamous cell carcinoma of the head and neck; TPEx, cisplatin, docetaxel, cetuximab. *Other first-line options include cetuximab + cisplatin, cetuximab + paclitaxel and other platinum-based treatments. ^†^Supported by phase 3 trial evidence.

Here, we review the clinical data and propose an optimal sequence of systemic therapies to maximize the continuum of care in R/M SCCHN based on currently available evidence (Figure 1). Non-systemic therapies (radiation therapy, surgery) are outside the scope of this manuscript and will not be discussed in detail.

## Available Targeted Therapies in SCCHN

### Cetuximab

**Figure 2:**
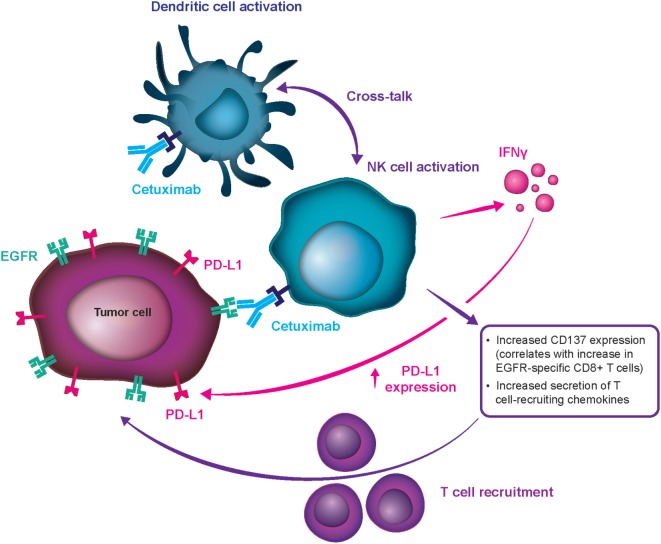
**Mechanism for cetuximab-mediated antibody-dependent cell-mediated cytotoxicity stimulation**. CD, clusters of differentiation; EGFR, epidermal growth factor receptor; IFN-γ, interferon-γ; NK, natural killer; PD-L1, programmed cell death ligand 1.

Cetuximab was the first targeted therapy approved in the first line for R/M SCCHN, conferring survival benefits in combination with platinum-based CT (7, 9, 19–21). SCCHN tumors are heavily influenced by dysregulation of the epidermal growth factor receptor (EGFR) pathway, and high EGFR expression is related to worse outcomes (22). Cetuximab is an immunoglobulin G subclass 1 (IgG1) monoclonal antibody (mAb) targeting the EGFR by preventing its ligand-mediated activation and dimerization, and it thus inhibits tumor cell proliferation and stimulates proapoptotic pathways within the tumor cell (23–25). Furthermore, cetuximab limits EGFR’s potential for translocation into the cell nucleus and leads to inhibition of double-stranded DNA break repair by preventing activation of the DNA-dependent protein kinase. This activity may also have an effect on pathways of tumor metastasis (26, 27). Finally, the IgG1 isotype allows cetuximab to induce antibody-dependent cell-mediated cytotoxicity (ADCC), which is the process of immune cells targeting and killing cells coated in IgG1 or other isotypes of antibodies (23, 28, 29). In addition to its apoptosis-inducing, EGFR-blocking activity, cetuximab directs the ADCC mechanism at tumor cells, using primarily natural killer (NK) cells to maximize antitumor effects and thereby representing the first immunotherapy in SCCHN (Figure 2) (20, 24, 30, 31).

Multiple preclinical studies have demonstrated cetuximab’s ability to stimulate ADCC and affect antitumor immunity. *In vitro* evidence shows that cetuximab can mobilize NK cells, activate neutrophils, and stimulate dendritic cell maturation (20, 23, 29, 30). Furthermore, cetuximab treatment results in an increase in cytotoxic CD8+ T cells in peripheral blood samples from patients with SCCHN receiving weekly cetuximab plus chemoradiotherapy during clinical trials (23). Finally, enhanced cytotoxic activity has been documented by *ex vivo* ADCC assays in patients with R/M SCCHN receiving cetuximab-based therapy, and induced ADCC was shown to be associated with positive clinical outcomes (20). Although it is challenging to prove in clinical studies, data suggest that this property may be involved in cetuximab’s antitumor activity in humans (23, 24, 29, 30, 32). It has also been proposed that stimulation of ADCC is an underlying mechanism for cetuximab’s clinically meaningful activity and the comparatively notable response rates with first- and second-line treatment in patients with R/M SCCHN, which set it apart from other mAb EGFR inhibitors (e.g., panitumumab) (33). Cetuximab can be distinguished from panitumumab in terms of their differential effect on the immune system. Panitumumab, an IgG2 antibody with weak ADCC action, had lower clinical activity as monotherapy (34) and in combination regimens (SPECTRUM, and other) (35) in R/M SCCHN. Therefore, the advantage in meaningful clinical activity of cetuximab over panitumumab in SCCHN may be partially attributed to their effects beyond EGFR inhibition (notably, panitumumab is a very potent EGFR inhibitor), i.e., to the differential induction of immune response, which seems to be highly relevant in SCCHN (Figure 2) but less relevant in colorectal cancer.

In SCCHN, a highly immunogenic disease, combinations of immunotherapies such as ICIs, antitumor vaccines, cetuximab (through ADCC action), and engineered T cells may have the potential to further improve standard response rates (31). It is also reasonable to propose that “priming” antitumor immune responses with cetuximab prior to the administration of other immunotherapies might augment a patient’s responsiveness to treatment (31, 36). Contribution of ADCC to the antitumor activity of cetuximab have not been widely tested in clinical trials, but recent studies has suggested a correlation between cetuximab efficacy and high ADCC (37, 38).

### Immune Checkpoint Inhibitors

ICIs are a new class of therapeutics in cancer. They function *via* the interruption of immunosuppressive pathways, called inhibitory checkpoints, which are normally used by tumor cells to prevent detection and elimination by the host immune system (31, 39). Molecular targets of ICIs found on T cells include cytotoxic T-lymphocyte antigen-4 (CTLA-4) and programmed cell death protein 1 (PD-1) receptor. A third common target is PD-1’s corresponding ligand, PD-L1, found on both tumor and immune cells (31, 39). ICIs are projected to be particularly successful in tumors with high levels of endogenous PD-L1 expression, including SCCHN (31). A full list of ICIs currently in advanced clinical trials for SCCHN can be found in Table 1, including anti-PD-1 antibodies nivolumab and pembrolizumab, as well as the anti-PD-L1 antibodies durvalumab and avelumab. Newer agents targeting immuno-inhibitory (VISTA, Tim-3, LAG3) or stimulatory (CD137, GITR, OX-40) molecules are also being studied (40). As of this writing, the bulk of available data on the efficacy of ICIs in SCCHN are derived from trials with platinum-refractory and second- or later-line R/M SCCHN patient populations predominantly with an Eastern Cooperative Oncology Group performance status (ECOG PS) of 0 or 1.

**Table 1:** **PD-1 axis immune checkpoint inhibitors under development for recurrent or metastatic squamous cell carcinoma of the head and neck**.

Name	Sponsor	Isotype	Target	Phase	Schedule used in clinical study	Regimen
Atezolizumab	Genentech	IgG1	PD-L1	1	0.01–20 mg/kg q3w	Monotherapy
				1/2	1,200 mg	Combination with anti-CD27
Avelumab	Merck KGaA	IgG1	PD-L1	1	1–20 mg/kg q2w (41)	Monotherapy
	Pfizer			1	N/A	Combination with anti-OX40
				1	N/A	Combination with anti-4-1BB
Durvalumab	MedImmune	IgG1	PD-L1	3	1,500 mg q4w (42)	Monotherapy
				3	1,500 mg q4w (42)	Combination with anti-CTLA-4
				1	N/A	Combination with anti-CTLA-4 and chemotherapy (CT)
Ipilimumab	Bristol-Myers Squibb	IgG1	CTLA-4	3	N/A	Combination with anti-PD-1
				1	3 mg/kg q3w (43)	Combination with anti-B7-H3
Nivolumab	Ono/Bristol-Myers Squibb	IgG4	PD-1	3	3 mg/kg q2w (44)	Monotherapy
				3	N/A	Combination with anti-CTLA-4
Pembrolizumab	MSD	IgG4	PD-1	3	200 mg q3w (45)	Monotherapy
				3	200 mg q3w (46)	Combination with CT
				1	2 mg/kg q3w (47)	Combination with anti-B7-H3
				2	2 mg/kg q3w	Combination with anti-EGFR
Tremelimumab	AstraZeneca	IgG2	CTLA-4	2	N/A	Monotherapy
				3	75 mg q4w (42)	Combination with anti-PD-L1
				1	N/A	Combination with anti-PD-L1 and CT

*CD, clusters of differentiation; CTLA-4, cytotoxic T-lymphocyte antigen-4; EGFR, epidermal growth factor receptor; IgG, immunoglobulin G; N/A, not available; PD-1, programmed cell death protein 1; PD-L1, programmed cell death ligand 1; q2w, every 2 weeks; q3w, every 3 weeks; q4w, every 4 weeks*.

## First-Line Treatment Options in R/M SCCHN

For decades prior to the introduction of the EXTREME regimen [platinum, 5-FU, cetuximab, followed by maintenance cetuximab until progressive disease (PD) or toxicity], no experimental treatments yielded any significant increase in survival in patients with R/M SCCHN.

EXTREME was a randomized phase 3 trial published in 2008, and the EXTREME regimen became the first to improve PFS and OS in patients with R/M SCCHN (Table 2) (9, 48). The study included fit patients (*n* = 442) with R/M SCCHN, of whom 88% had ECOG PS 0–1/Karnofsky score ≥80 and 12% had ECOG PS 2/Karnofsky score <80, who were ineligible for local therapy. The main exclusion criteria were surgery or irradiation within the previous 4 weeks, or previous systemic therapy unless it was part of a multimodal treatment for LA disease that had been completed >6 months before study entry (9). The trial investigated whether the addition of cetuximab to platinum-based CT with cisplatin or carboplatin plus 5-FU followed by maintenance cetuximab until PD or toxicity in the first line would improve survival in patients with R/M SCCHN. The primary endpoint of the EXTREME study was OS, which it met as median OS was significantly improved from 7.4 to 10.1 months [hazard ratio (HR) = 0.80 (95% CI, 0.64–0.99)] in the CT and cetuximab plus CT arms, respectively (9). Median PFS was also significantly improved by the addition of cetuximab to first-line CT, from 3.3 to 5.6 months [HR = 0.54 (95% CI, 0.43–0.67)] (9). The ORR in patients with R/M SCCHN was nearly doubled upon the addition of cetuximab to CT [36 vs 20%; odds ratio = 2.33 (95% CI, 1.50–3.60)] (9). Finally, the addition of cetuximab to platinum-based CT led to a significant reduction in pain and in problems with social eating and speech, thereby positively impacting social functioning and quality of life for patients receiving this treatment (49). The toxicity profile of the EXTREME regimen has been shown to be predictable and manageable (9).

**Table 2:** **Cetuximab-based therapy options for first-line treatment of recurrent or metastatic squamous cell carcinoma of the head and neck**.

Name	Regimen	Median PFS, months	Median OS, months	ORR, %	Dosage details
EXTREME (*n* = 442)	Cetuximab + cisplatin/carboplatin + 5-FU	5.6	10.1	36	Cisplatin (100 mg/m^2^ on day 1) or carboplatin (AUC 5 mg/mL/min, as a 1-h intravenous infusion on day 1) and 5-FU (1,000 mg/m^2^ per day for 4 days) every 3 weeks for a maximum of 6 cycles Cetuximab (initial dose of 400 mg/m^2^ as a 2-h intravenous infusion, then 250 mg/m^2^ as a 1-h intravenous infusion per week) for a maximum of 6 cycles
PCE (*n* = 45)	Cetuximab + carboplatin + paclitaxel	5.2	14.7	40	Paclitaxel (100 mg/m^2^ on day 1 and 8) and carboplatin (AUC 2.5 on day 1 and 8), repeated every 3 weeks for up to 6 cycles Cetuximab (initial dose of 400 mg/m^2^, followed by 250 mg/m^2^ weekly) until PD or unacceptable toxicities
TPEx (*n* = 54)	Cetuximab + cisplatin + docetaxel	6.2	14	44.4	Docetaxel and cisplatin (75 mg/m^2^ both) on day 1 Weekly cetuximab 250 mg/m^2^ (initial dose of 400 mg/m^2^) Treatment was repeated every 21 days for 4 cycles, followed by maintenance cetuximab (500 mg/m^2^) every 2 weeks until PD or unacceptable toxicity
Burtness et al. (50) (*n* = 117)	Cetuximab + cisplatin	4.2	9.2	26	Cetuximab was given (dose of 200 mL/m^2^) intravenously on day 1 over 120 min for 1 cycle only; subsequent cycles were administered at 125 mL/m^2^/week intravenously over 60 min Cisplatin (100 mg/m^2^) was given on day 1 every 4 weeks
Hitt et al. (51) (*n* = 46)	Cetuximab + paclitaxel	4.2	8.1	54	Paclitaxel (80 mg/m^2^) and cetuximab (initial dose 400 mg/m^2^, subsequent doses of 250 mg/m^2^) were given weekly until PD or unacceptable toxicity

*5-FU, 5-fluorouracil; AUC, area under the curve; ORR, overall response rate; OS, overall survival; PD, progressive disease; PFS, progression-free survival*.

It is important to note that patients eligible for the EXTREME regimen in first-line R/M SCCHN are not necessarily treatment-naïve and may have received previous platinum-containing therapy (but ≥6 months previously) in the LA SCCHN setting (9, 52–54). The role of cetuximab in first-line R/M SCCHN specifically for patients who have previously received cetuximab for LA SCCHN has not been fully investigated, although responsiveness to retreatment in such patients has been documented (55). In addition, the importance of polychemotherapy in combination with cetuximab is suggested when considering data from the randomized trial by Burtness et al., in which cetuximab plus cisplatin showed improved activity in first-line R/M SCCHN (ORR was ≥2.5-fold higher in the cetuximab plus cisplatin arm), but did not show statistically significant OS benefits compared with cisplatin monotherapy. The results of this trial, however, are difficult to interpret definitively due to the small sample size (*n* = 117 patients) (50).

Over the past decade, retrospective and observational studies have consistently confirmed the benefits of the EXTREME regimen in patients with first-line R/M SCCHN (9, 11, 56). In the DIRECT study, prospective data were collected for 154 patients with untreated SCCHN in the R/M setting receiving cetuximab according to the EXTREME regimen guidelines (53). The EXTREME regimen was shown to be feasible in everyday clinical practice and yielded a median time to progression of 6 months, which is in line with PFS findings in the EXTREME study (9, 11, 56). Siano et al. have also shown benefits of such regimens in a retrospective analysis of 117 patients treated with cetuximab plus platinum-based CT with or without 5-FU in first-line R/M SCCHN (57). The median OS of 12.4 months was in line with OS findings for cetuximab plus platinum-based CT in clinical trials (9, 11, 56). Median PFS and OS during follow-up second-line treatment with methotrexate, paclitaxel, or other agents were between 2.6 and 6.1 months, showing the need for stronger options in later-line treatments of R/M SCCHN. De Mello et al. also conducted a retrospective study of 121 patients receiving the EXTREME regimen as first-line treatment in the R/M SCCHN setting (58). Median PFS and OS were 8 and 11 months, respectively, which are also in line with findings for cetuximab plus platinum-based CT in clinical trials (9, 11, 56). Similarly, in a retrospective study of 31 patients with R/M SCCHN, cetuximab plus CT followed by maintenance with biweekly cetuximab led to a stable disease rate of 52%, partial response rate of 39%, and complete response rate of 9% (59). Furthermore, there is evidence that maintenance therapy until PD with cetuximab as a single agent following the EXTREME regimen is also well-tolerated with a good compliance (relative dose intensity = 82%) (53, 59). Indeed, in the EXTREME trial, the frequency of severe skin reactions in the cetuximab-containing arm decreased from 9 to 5% during the cetuximab maintenance phase (median treatment duration = 29.9 weeks) (60).

It is important to point out that in SPECTRUM, an analogous phase 3 study to the EXTREME trial, the addition of the anti-EGFR mAb panitumumab to cisplatin and 5-FU yielded a statistically significant improvement in PFS (5.8 vs 4.6 months) and ORR (36 vs 25%), but not in OS (35). These data confirm the utility of anti-EGFR therapy in first-line treatment of R/M SCCHN, while also suggesting that cetuximab and panitumumab, an IgG1 and an IgG2 mAb, respectively, do not produce identical survival outcomes in patients with SCCHN. As previously discussed, these differential outcomes could be partially attributed to the distinct property of ADCC stimulation by cetuximab (Figure 2) that potentially enhances antitumor activity in SCCHN.

### Using a Taxane Instead of 5-FU in the Backbone Regimen

Chemotherapy backbones for the EXTREME regimen can be altered according to patient needs by substituting a taxane for 5-FU to boost response rates and/or circumvent contraindications to 5-FU (61). Details on regimens and doses are outlined in Table 2. The TPE (cisplatin, docetaxel, cetuximab) combination regimen was originally introduced by Argiris et al. in a phase 2 trial in LA SCCHN (62) and was subsequently investigated in the phase 2 GORTE C 2008-03 study (the so-called “TPEx” regimen) in the first-line treatment of R/M SCCHN (56). After four cycles of TPEx, patients without PD received maintenance cetuximab every 2 weeks until PD. The GORTEC 2008-03 study included a similar patient population to the EXTREME study, i.e., patients with previously untreated R/M SCCHN, except for any treatment received in the LA setting ≥6 months prior to study entry. ORR in patients who received TPEx was 44.4%, and median OS was 14 months. The PCE regimen similarly consists of paclitaxel, carboplatin, and cetuximab followed by cetuximab maintenance until PD or toxicity (Table 2) (11). A trial of PCE, which was presented at the 2016 annual meeting of the American Society of Clinical Oncology (ASCO), also included patients who were EXTREME eligible and received PCE as first-line treatment for R/M SCCHN. ORR was 40% and the median OS was 14.7 months. Finally, a phase 2 study found that cetuximab plus weekly paclitaxel given in the first line yielded an ORR of 54% and 10 complete responses (22% of the patient population of the trial) (51). Overall, cetuximab plus CT-based treatments for first-line R/M SCCHN are associated with high ORRs, extending OS and permitting a large number of patients to achieve disease control. Therefore, in cases of PD on this first-line treatment, a high number of patients have been able to enter second-line therapy. Finally, it is important to note that an ongoing phase 2 trial led by GORTEC is comparing TPEx with EXTREME in the first-line treatment of R/M SCCHN (NCT02268695), with OS as the primary endpoint and ORR, PFS, and safety as secondary outcomes. The results of this trial will prove very informative to oncologists when deciding which regimen is more appropriate for their patients, except in clear cases where the patient has a contraindication to 5-FU.

### Management of Unfit Patients

Although a patient with an ECOG PS of 0 or 1 would receive the EXTREME regimen in the first line, a patient with an ECOG PS of 2 would generally be placed on a single-agent therapy in the first line, including, but not limited to, targeted therapeutics such as cetuximab or cytotoxic CT agents such as methotrexate, docetaxel, paclitaxel, carboplatin, 5-FU, and capecitabine (9, 52, 54, 61). Another option may be the combination of cetuximab with a taxane for selected patients. However, there are no data showing benefit from any of these treatments (monotherapy or combination) in a controlled, randomized trial. For example, a phase 3 trial that compared gefitinib with methotrexate (the IMEX trial) and another that compared docetaxel with or without gefitinib, which both enrolled some patients with an ECOG PS of 2, failed to show survival benefits with these newer therapies over standard monotherapy with methotrexate or a taxane (63, 64). Zalutumumab, an anti-EGFR antibody, did not result in prolonged OS compared with best supportive care (BSC) alone (many patients received methotrexate in the control arm) in a patient population with platinum-refractory R/M SCCHN, which included patients with an ECOG PS of 2 (16). First-line cetuximab vs methotrexate monotherapy is currently being evaluated in the unfit elderly (≥70 years of age) with R/M SCCHN in a randomized phase 3 trial (ELAN-UNFIT, NCT01884623).

The use of ICI monotherapy for the treatment of unfit patients is of interest and deserves clinical study. Given their preferable toxicity profile and documented activity in SCCHN, we propose that ICIs can be potentially incorporated in the therapeutic algorithm (Figure 1). However, it should be recognized that the activity of ICIs as monotherapy is low and the majority of patients progress at first reevaluation; therefore, symptomatic patients with PS of 2 due to disease may deteriorate rapidly.

### Status of ICI Clinical Trials for First-Line Treatment

Immune checkpoint inhibitors such as nivolumab, pembrolizumab, and durvalumab are currently being investigated in the first-line R/M SCCHN setting for fit patients, with the EXTREME regimen chosen as a comparator arm in several recently opened phase 3 trials (Table 3). Nivolumab in combination with the anti-CTLA-4 antibody ipilimumab is compared with the EXTREME regimen in the CheckMate 651 trial. In contrast, pembrolizumab is being investigated as a monotherapy as well as in combination with CT for first-line R/M SCCHN (KEYNOTE-048). Finally, durvalumab is under examination as a monotherapy and in combination with the anti-CTLA-4 antibody tremelimumab (the KESTREL study). However, no results are yet available from these trials as of the time of this writing and, therefore, use of ICIs in first-line R/M setting is not recommended outside of clinical trials (13, 14, 42, 46, 65, 66). Finally, it is worth noting that a recent trial showed no improvement in outcomes by adding an immunotherapeutic agent (motolimod) to the EXTREME regimen as quadruplet therapy in first-line R/M SCCHN (67).

**Table 3:** **Ongoing studies with immune checkpoint inhibitors in first-line R/M SCCHN vs standard of care (EXTREME regimen)**.

NCT #	Immunotherapy agent(s) in study	Phase	Population	Arms
NCT02741570 (CheckMate 651)	Nivolumab, ipilimumab	3	Previously untreated R/M SCCHN, ≥6 months since last dose of platinum	Nivolumab + ipilimumab vs EXTREME
NCT02358031 (KEYNOTE-048)	Pembrolizumab	3		Pembrolizumab vs Pembrolizumab + CT vs EXTREME
NCT02551159 (KESTREL)	Durvalumab, Tremelimumab	3		Durvalumab vs Durvalumab + tremelimumab vs EXTREME

*CT, chemotherapy; EXTREME, cetuximab plus cisplatin/carboplatin plus 5-fluorouracil followed by maintenance cetuximab; NCT, ClinicalTrials.gov identifier; R/M SCCHN, recurrent or metastatic squamous cell carcinoma of the head and neck*.

## Second-Line and Platinum-Refractory Options in R/M SCCHN

Over the past decade, patients with second-line R/M SCCHN predominantly received either single-agent CT or BSC, or they entered clinical trials (52, 54). Pembrolizumab and nivolumab were granted US Food and Drug Administration approval in 2016 (August and November, respectively) for use in patients with R/M SCCHN who progress on or after platinum-based CT, with no PD-L1 testing requirement in place.

For the purposes of this review (and based on patient selection criteria for these trials), fit patients who progress on EXTREME first-line therapy in the R/M setting will be considered as entering second- or later-line therapy for R/M SCCHN (52, 54). Fit patients who progress within 6 months after the last administered dose of platinum (cisplatin or carboplatin) in either the LA or R/M setting are, due to this very short duration since the last platinum treatment, not optimal candidates for platinum retreatment. These patients have been referred to as “platinum-refractory” (i.e., also EXTREME-ineligible). Treatment options are very similar between patients with platinum-refractory disease and those in second-line R/M SCCHN and, therefore, these two patient subgroups will be discussed together here and labeled as “second-line” (68–70). Moreover, most of the second-line trials in R/M SCCHN have traditionally allowed any number of prior therapies for R/M disease and, therefore, in these trials, “second-line” therapy implies “second-line and beyond.” Although not the principal focus of this review manuscript, we will also briefly discuss recent observations involving kinase inhibitors such as afatinib (a pan-human EGFR inhibitor, which blocks tyrosine kinase function in human EGFR 1, 2, and 4) and buparlisib [targeting phosphoinositol-3-kinase (PI3K)].

### Chemotherapy

Available agents for second-line therapy in R/M SCCHN include methotrexate, docetaxel, and paclitaxel. ORR to methotrexate monotherapy in the second line is 6%, and median OS is ≈6.0 months (15, 63). Second-line treatment with a taxane, such as docetaxel or paclitaxel, is frequently used in R/M SCCHN but has not been demonstrated to be superior to other agents in this setting (16, 61, 64). Additionally, while the CheckMate 141 trial was not designed to compare the three regimens used in the comparator arm, docetaxel appeared slightly and numerically superior to methotrexate (and possibly cetuximab, although only 15 patients received this treatment) in terms of OS, although no concrete conclusions can be drawn, and phase 2 randomized trials suggest no difference in survival between these monotherapies (44, 71). Irinotecan has also shown some very limited activity in this setting (7). Combination therapy does not appear to yield better results than monotherapy as a second-line treatment for patients with R/M SCCHN. A phase 2 trial of irinotecan and docetaxel that enrolled patients with good performance status in second-line treatment showed poor results with an objective response rate of 3% and a median survival of 5 months (70). Patients who received BSC for the sole purpose of symptom management also attained a median OS of ≈5 months (16).

### Anti-EGFR Therapy

Retreatment with platinum in the setting of platinum-refractory disease increased toxicity without improving efficacy outcomes (5, 72). Cetuximab plus cisplatin or carboplatin in platinum-refractory patients within 50 days of the last platinum dose of the previous regimen in the R/M setting achieved ORRs of 10% with a median OS of ≈6 months (5). Herbst et al. reported an ORR to cetuximab plus cisplatin of 6% and a median OS of 4.3 months in patients with platinum-refractory disease if PD occurred within 90 days of the last platinum dose prior to entering the study (73).

Cetuximab monotherapy in second-line and platinum-refractory R/M SCCHN populations has been tested in 3 phase 2 clinical trials by Vermorken et al., Baselga et al., and Herbst et al., involving patients who had progressed on cisplatin- or carboplatin-based regimens (5, 12, 72, 73). ORRs of 10–13% were observed. Disease control rates while on cetuximab monotherapy can top 50%, with median OS of between 5 and 6 months (7, 12, 61, 72). Currently, cetuximab monotherapy is approved in the US but has not been compared with BSC as of yet.

Finally, as in the first line, cetuximab plus paclitaxel is also a palliative option in the second-line setting, offering ORRs of 38–55% and median OS of 7.6–10 months (74–76). Cetuximab plus docetaxel given to patients with R/M SCCHN after failure of platinum-based therapy resulted in 11% of patients achieving partial responses and 40% achieving stable disease. Disease control rates were similar between patients with platinum-sensitive and platinum-refractory disease, and median OS was 6.7 months (77).

Other anti-EGFR therapies have failed to offer higher efficacy results. Second-line panitumumab monotherapy yielded an ORR of 4% and a median OS of 5.1 months in the PRISM study (34). Non-mAb EGFR tyrosine kinase inhibitors such as gefitinib, erlotinib, lapatinib, and afatinib have also yielded results that must be regarded as modest at best. In a randomized phase 3 trial, patients who had progressed on or were not fit for standard first-line therapy received docetaxel plus a placebo or docetaxel plus gefitinib. ORRs for the docetaxel plus placebo vs docetaxel plus gefitinib arms were 6.2 and 12.5%, respectively, with a non-significant improvement in median OS of 1.3 months (from 6.0 to 7.3 months). Similar results have been obtained from the LUX-Head and Neck 1 trial with afatinib. LUX1 was a randomized phase 3 trial comparing afatinib vs methotrexate in fit patients with R/M SCCHN who had progressed on or after first-line platinum-based therapy. When compared with the efficacy of methotrexate monotherapy described above, afatinib yielded a median OS of 6.8 months (no improvement), with an ORR of 10% (15, 63, 64). Therefore, afatinib has no survival advantage over methotrexate in this setting and cannot be considered as an evidence-based approach in second-line therapy. Another phase 2 trial showed comparable efficacy with some toxicity profile differences between cetuximab and afatinib (78). Whether selected patients may benefit from afatinib (e.g., those with p16-negative disease) has yet to be demonstrated in prospective clinical trials (79).

### PI3K Inhibition

Advances are being made in the use of PI3K inhibition in second- or later-line R/M SCCHN therapy. Because the PI3K/Akt/mTOR pathway is a signaling cascade downstream of the EGFR that stimulates cell growth and is often dysregulated in tumor cells, targeting this pathway is one potential method for overcoming resistance to anti-EGFR targeted therapy (80). The BERIL-1 trial of the pan-PI3K inhibitor buparlisib (BKM120) in second- and third-line settings was presented at ASCO 2016. Buparlisib plus paclitaxel treatment was compared to a placebo plus paclitaxel regimen in platinum-pretreated patients with one or two previous lines of therapy in the R/M SCCHN setting. The response rate to buparlisib plus paclitaxel was 39% (vs 14% in the comparator arm), with patients achieving a median OS of 10.0 months on this treatment (vs 6.5 months; no indicator of statistical significance was presented) (81).

### ICI Therapy

Currently, ICIs present an efficacious therapeutic option for patients with R/M SCCHN who have progressed after platinum-based therapy, which supports the emergence of a continuum of care for R/M disease (Figure 1).

Pembrolizumab and nivolumab approvals in the US for patients with R/M SCCHN who progress on or after platinum-containing treatment were based on results from the KEYNOTE-012 and CheckMate 141 trials. KEYNOTE-012, a non-randomized phase 1b trial with pembrolizumab monotherapy, enrolled 192 patients. The first 60 patients (“Cohort B”) were selected for PD-L1–positive tumors and treated with 10 mg/kg pembrolizumab intravenously every 2 weeks, and the remaining 132 patients (“Cohort B2”) were unselected for tumor PD-L1 expression and treated with pembrolizumab at a fixed dose of 200 mg intravenously every 3 weeks (13, 45, 82). Among Cohort B patients (i.e., patients with PD-L1-positive tumors), 70% had received ≥2 lines of therapy (13). For this cohort, median PFS and OS were 2 and 13 months, respectively. In Cohort B2, i.e., patients unselected for PD-L1 expression status, 18% had not received prior therapy in the R/M setting, while 57% had received ≥2 lines of therapy (45). These patients achieved a median PFS of 2 months and median OS of 8 months, with tolerable safety. The observed ORR was 18% in both cohorts (45, 82).

CheckMate 141 was a phase 3 trial that enrolled 361 patients with R/M SCCHN, of any tumor PD-L1 expression status, who had disease progression within 6 months after platinum-based CT (Table 4) (44). Nivolumab 3 mg/kg every 2 weeks was compared to the investigator’s choice (IC) of standard therapy (methotrexate, docetaxel, or cetuximab). Nivolumab monotherapy yielded superior OS over standard therapy with a median OS of 7.5 months (vs 5.1 months with standard therapy) with an HR for death, 0.70 (*p* = 0.01). However, the median PFS was similar in the two arms (2 months with nivolumab and 2.3 months with standard therapy, HR for disease progression or death, 0.89; *p* = 0.32) and the ORR was 13.3% with nivolumab vs 5.8% with standard therapy (44). Notably, at 6 months, the rate of PFS was 19.7% with nivolumab vs 9.9% with standard therapy, whereas at 1 year, the rate of OS was 36% with nivolumab vs 16.6% with standard therapy.

**Table 4:** **Studies with immune checkpoint inhibitors in mixed-, second-, and later-line settings and platinum-refractory R/M SCCHN**.

NCT #	Drug	Phase	Population	Arms
NCT02105636 (CheckMate 141)	Nivolumab	3 (completed)	Platinum-refractory Second-/later-line R/M SCCHN	Nivolumab vs IC (cetuximab or docetaxel or methotrexate)
NCT01848834 (KEYNOTE-012)	Pembrolizumab	1b	Previously untreated R/M SCCHN, ≥6 months since last dose of platinum Platinum-refractory Second-/later-line R/M SCCHN	Pembrolizumab monotherapy
NCT02255097^a^ (KEYNOTE-055)	Pembrolizumab	2	Platinum-refractory Second-/later-line R/M SCCHN	Pembrolizumab monotherapy
NCT02252042 (KEYNOTE-040)	Pembrolizumab	3	Platinum-refractory Second-/later-line R/M SCCHN	Pembrolizumab vs IC (cetuximab or docetaxel or methotrexate)
NCT02823574 (CheckMate 714)^b^	Nivolumab, ipilimumab	2	Previously untreated R/M SCCHN, ≥6 months since last dose of platinum Platinum-refractory	Nivolumab + ipilimumab vs Nivolumab + placebo
NCT02369874 (EAGLE)	Durvalumab	3	Platinum-refractory Second-/later-line R/M SCCHN	Durvalumab vs Durvalumab + tremelimumab vs IC (fluoropyrimidine, cetuximab, taxane, or methotrexate)
	tremelimumab			
NCT02207530 (HAWK)	Durvalumab	2	Platinum-refractory Second-/later-line R/M SCCHN	Durvalumab monotherapy

*IC, investigator’s choice; NCT, ClinicalTrials.gov identifier; R/M SCCHN, recurrent and/or metastatic squamous cell carcinoma of the head and neck*.

*^a^All patients in KEYNOTE-055 were also cetuximab-refractory*.

*^b^CheckMate 714 enrolls a cohort of patients in the first-line setting*.

Safety findings from ICI studies with available data indicated a favorable and tolerable toxicity profile for pembrolizumab and nivolumab over standard therapies in second-line and platinum-refractory R/M SCCHN (Table 5). Treatment-related events occurred in 58.9% (grade 3–4 in 13.1%) and 62% (grade 3–4 in 9%) of patients in CheckMate 141 and KEYNOTE-012, respectively (44, 45). Finally, patient-reported outcomes from CheckMate 141 revealed stabilization or slight improvement in quality of life measures such as social functioning and pain, while IC monochemotherapy resulted in a clinically meaningful worsening across many of the same measures (44).

**Table 5:** **Available data for immune checkpoint inhibitor monotherapy in recurrent or metastatic squamous cell carcinoma of the head and neck (14, 67, 80)**.^a^

NCT #	No. of Patients	Eligibility	Drug	Available data			Toxicity findings
				Median PFS, months	Median OS, months	ORR, %	
NCT02105636 (CheckMate 141) (67)	361		Nivolumab	2	7.5	13.3	13.1% of patients experienced grade 3–4 TRAEs
							2 patients died due to TRAE (1 pneumonitis and 1 hypercalcemia)
NCT01848834 (expanded KEYNOTE-012) (80)	60	PD-L1+	Pembrolizumab	2	13	18	17% of patients experienced grade 3–4 TRAEs
	132	Any PD-L1 status		2	8	18	No treatment-related deaths were reported
							About 9% of patients experienced grade 3–4 TRAEs
							No treatment-related deaths were reported
NCT02255097 (KEYNOTE-055) (14)	171		Pembrolizumab	2.1	8	16	26 patients (15%) experienced grade 3–5 TRAEs
							1 patient died of treatment-related pneumonitis

*NCT, ClinicalTrials.gov identifier; ORR, overall response rate; OS, overall survival; PD-L1, programmed cell death ligand 1; PFS, progression-free survival; TRAE, treatment-related adverse event*.

*^a^The values in this table reflect the PFS, OS, and ORR recorded for the populations of each trial regardless of PD-L1 expression status (14, 67, 80)*.

Although ICIs may not improve median PFS, as compared to IC therapy (Table 5) (13, 14, 31, 65), the subset of patients who achieve a response or stable disease on ICI treatment tend to experience a longer duration of response and OS than is to be expected with standard CT (14, 82, 83). For patients with SCCHN, ORRs to ICI monotherapy range from 13 to 18% (Table 4). Somewhat higher tumor responses and survival are seen with positive tumor PD-L1 expression status even though patients with PD-L1-negative tumors may also benefit (84).

Other trials of ICI monotherapy are also tackling second-line R/M SCCHN (patient inclusion criteria in Table 4 and full data summary in Table 5). Ongoing trials with pembrolizumab include KEYNOTE-040 and MASTERKEY232 (combination pembrolizumab and talimogene laherparepvec in second-line and platinum-refractory R/M SCCHN). Upcoming nivolumab trials in SCCHN include CheckMate 714 (mixed population of patients with platinum-refractory and first-line R/M SCCHN). Finally, ongoing trials with durvalumab include the phase 2 HAWK study (monotherapy in second-line, PD-L1-positive R/M SCCHN following a single platinum-based treatment), and the phase 3 EAGLE study (monotherapy or combination with tremelimumab in second-line R/M SCCHN following a single platinum-based treatment) (14, 65, 66, 85, 86).

### Patient Selection for ICI Therapy: PD-L1 and p16/Human Papillomavirus (HPV) Status and Other Biomarkers

Based on the available clinical trial-generated evidence, ICIs present a promising new opportunity for a small number of patients with very advanced disease who progress on standard therapy. ORRs to ICIs remain around the 20% mark in second-line patients with R/M SCCHN unselected for tumor PD-L1 expression status. Although data continue to suggest that PD-L1 expression correlates with better efficacy to ICIs, the correlation is not definitive and no other reliable biomarker for effectively selecting optimally responsive patient subgroups has been identified as of yet (31, 83). Until additional reliable biomarkers for the efficacy of PD-1/PD-L1 blockade are identified, patient selection for pembrolizumab, durvalumab, and nivolumab therapy remains unsatisfactory. In the CheckMate 141 trial, positive tumor PD-L1 expression appeared to have conferred a numerically higher survival benefit in patients receiving nivolumab vs IC therapy [HR = 0.55 (95% CI, 0.36–0.83) in patients with tumors expressing ≥1% PD-L1 vs HR = 0.89 (95% CI, 0.54–1.45) in patients with tumors expressing <1% PD-L1] (44, 65). However, it is important to note that the trial was not powered to detect interactions between tumor PD-L1 expression status and treatment. Subgroup analyses of KEYNOTE-012 also indicated higher OS and ORR in patients with ≥1% PD-L1-expressing tumors who had received ≥1 dose of pembrolizumab (45, 87). When immune cells were included in the PD-L1 expression analysis, ORR rose to 22% in patients with PD-L1-expressing tumors but dropped to 4% in those with <1% PD-L1 expression (45). The predictive value of tumor PD-L1 expression continues to be investigated. Prospective trial populations will need to be large enough to detect such an interaction because the sample sizes in trials completed to this date have been relatively small. Furthermore, there are currently multiple methodologies available for assessing PD-L1 positivity and some controversy exists about which cells should be included in such an analysis (88). Future trials will hopefully educate on these topics as well. However, it appears that treating patients with ICIs irrespective of PD-L1 expression status may subject a larger number of likely non-responders to non-optimal therapy. At the same time, introducing a minimum PD-L1 expression requirement will ostensibly preclude a number of responders from receiving potentially efficacious ICI therapy, which becomes especially important in the second-line and platinum-refractory setting where there are few other efficacious treatments.

Additionally, the prognostic value of HPV status appears to hold up in patients treated with ICIs. In CheckMate 141, patients with p16-positive tumors experienced a numerically larger magnitude of benefit from nivolumab treatment as compared to patients with p16-negative tumors in terms of OS [HR (95% CI) = 0.56 (0.32, 0.99) for patients with p16-positive tumors vs HR (95% CI) = 0.73 (0.42, 1.25) for patients with p16-negative tumors] (65). Subgroup analyses of response rates to pembrolizumab by HPV status revealed that the ORR in patients with HPV-positive disease was 22 vs 16% in patients with HPV-negative disease in the KEYNOTE-055 study, and 32 vs 14%, respectively, in the KEYNOTE-012 study (14, 45). However, neither the effects of tumor PD-L1 expression nor HPV status are sufficiently robust in guiding the use of ICI therapy at this time. The analysis of future randomized trials will be very important in that regard.

Another potential biomarker of response to ICIs is the interferon-γ (IFNγ) 6-gene signature [*CXCL9* (C-X-C motif chemokine ligand 9), *CXCL10* (C-X-C motif chemokine ligand 10), *IDO1* (indoleamine 2,3-dioxygenase 1), *IFNG* (IFN-γ), *HLA-DRA* (major histocompatibility complex, class II, DR α), and *STAT1* (signal transducer and activator of transcription 1)] discussed by Chow et al. in the context of the KEYNOTE-012 study. In this analysis, a higher IFN-γ 6-gene score was significantly associated with response to pembrolizumab monotherapy and correlated with longer PFS and OS. The 6-gene score was not found to correlate in any way with HPV status (87).

Overall, ways for better patient selection for ICI therapy continue to be the subject of ongoing investigations. Nevertheless, the survival benefits for the subset of patients with pretreated R/M SCCHN who achieve disease control on ICI monotherapy are impressive and a breakthrough in clinical practice.

## A New Continuum of Care

Current clinical and observational evidence in the field supports the EXTREME regimen as standard of care for fit patients with R/M SCCHN in the first line, followed by the new treatment option of ICIs in second line (Figure 1). Maximizing the number of therapy lines and optimizing the order in which therapies are administered has historically been one of the most powerful tools for delivering maximum benefit to the greatest number of patients (17, 18). Until recently, this strategy has not been feasible in the treatment of R/M SCCHN; however, the emergence of ICIs has provided new options for an optimized continuum of care.

The majority of patients who do not respond during first-line treatments deteriorate rapidly; therefore, providing a continuum of care successfully requires maximizing the number of responses in first-line while patients are still relatively fit, thus allowing them to continue onto a second or third line of treatment. Indeed, responses are very important in patients with symptomatic disease, especially those with locoregional progression that can have devastating consequences. As the efficacy and safety of the EXTREME regimen are reproducible in observational studies, physicians’ decision-making when selecting and sequencing suitable therapies for patients with R/M SCCHN clearly pays off in clinical practice (57). Presently, there are no efficacy data for the reverse sequence (i.e., first-line ICIs followed by second-line utilization of the EXTREME regimen), or for robust follow-up therapeutic options for patients who progress on ICI treatment. Finally, platinum remains an important treatment component in SCCHN, and failure to treat with platinum-based CT in the first line could deprive patients of a line of therapy (5, 9, 52, 54, 73). Notably, our proposed sequence of treatments circumvents these potential limitations. In summary, until data for ICI function in first-line R/M SCCHN are published, the treatment sequence of first-line EXTREME regimen followed by second-line ICIs is the only evidence-based approach, and thus off-label use of ICIs in the first-line treatment of fit patients should be discouraged for the time being.

There are also additional unknowns, stemming from the relative novelty of ICI therapy, that further complicate the therapy selection process. For example, although ICIs confer OS benefits as compared to the rest of the available second-, later-line, and platinum-refractory setting therapeutics, there is still a clear need for biomarkers of response in order to improve patient selection, boost ORRs, and prolong PFS time (13, 14, 65, 82). Other observations associated with ICI monotherapy, including accelerated tumor growth rate (hyperprogression) in up to 9% of patients with various tumor types (89) and low median PFS in R/M SCCHN (44, 45, 82), also suggest that this treatment option is not yet ready to be administered in the first-line setting without supporting data from prospective clinical trials. Indeed, the use of first-line ICIs outside of a clinical trial (i.e., non-evidence-based use) may subject patients to the risk of early progression with worsening symptomatology and performance status, and a lower likelihood of staying sufficiently fit to receive additional therapy. Therefore, as the EXTREME and similar regimens result in 80% disease control rates with a median PFS of ≥5 months, these treatment options should continue to precede ICIs in the continuum of care for fit patients with R/M SCCHN.

## Conclusion

Treatment paradigms in R/M SCCHN are currently undergoing unprecedented evolution. Although the EXTREME regimen remains the evidence-based standard of care for first-line treatment of patients with R/M SCCHN, ICIs have shown promising OS results as monotherapies with tolerable toxicities and improvements in patient-reported outcomes in patients with R/M SCCHN in the second-, later-line, and platinum-refractory setting. Although the full extent of ICI functionality in different tumor types and therapy lines is still being discovered, we nevertheless anticipate that the pool of ICI-eligible patients will expand as data become available. The EXTREME regimen is supported by over 10 years of evidence, and its role in R/M SCCHN has been fully defined through years of clinical trials and observational studies. Given the impressive efficacy of ICIs in pretreated R/M SCCHN, the new challenge facing physicians is deciphering how to sequence available therapies optimally to maximally prolong patient survival, while maintaining the highest possible quality of life. Presently, the only evidence-based sequence places ICIs in the second-line and platinum-refractory settings, where they offer a promising alternative to historic therapeutic options. Therefore, fit patients with R/M SCCHN should continue to receive the EXTREME regimen in the first-line setting with cetuximab until PD. Quite excitingly, for the first time in a decade, physicians are able to offer patients with R/M SCCHN a continuum of care with efficacious therapy in multiple lines. Naturally, this treatment paradigm may evolve as additional data emerge.

## Author Contributions

All authors contributed equally to the conception of the intellectual content, interpretation of the data, and writing of the manuscript. All authors also reviewed any revisions that were made and provided their final approval of the manuscript.

## Conflict of Interest Statement

AA received honoraria from Bristol-Myers Squibb, Merck KGaA, Novartis, and Roche. KH has served as a consultant or advisor for and received honoraria from Amgen, AstraZeneca, Merck KGaA, and Pfizer; he has received research funding from all of the above companies and Boehringer-Ingelheim. MT received honoraria from Bayer, Bristol-Myers Squibb, Eisai, Merck Serono, and Otsuka, and served as a consultant or advisor to Bayer, MSD Pharmaceuticals, Ono Pharmaceutical, and Pfizer. MT has also received research funding from AstraZeneca, Bayer, Boehringer Ingelheim, Eisai, MSD, Novartis, NanoCarrier, Ono Pharmaceutical, and Pfizer. JS is an employee of Merck KGaA. PC is an employee of Merck KGaA. AC served as a consultant or advisor in exchange for honoraria and research funding from Amgen, AstraZeneca, Bayer, Bristol-Myers Squibb, Merck KGaA, and MSD Pharmaceuticals. LL served as a consultant or advisor for and received research funding from AstraZeneca, Bayer, Boehringer-Ingelheim, Bristol-Myers Squibb, Debiopharm, Eisai, Merck Serono, MSD Pharmaceuticals, Novartis, Roche, and Sobi; she has also received travel compensation from Bayer, Debiopharm, Merck Serono, and Sobi.

## Abbreviations

5-FU, 5-fluorouracil; ADCC, antibody-dependent cell-mediated cytotoxicity; BSC, best supportive care; CT, chemotherapy; CTLA-4, cytotoxic T-lymphocyte antigen-4; ECOG PS, Eastern Cooperative Oncology Group performance status; EGFR, epidermal growth factor receptor; EXTREME, cetuximab plus cisplatin/carboplatin plus 5-fluorouracil followed by maintenance cetuximab; HPV, human papillomavirus; IC, investigator’s choice; ICI, immune checkpoint inhibitor; IgG, immunoglobulin G; LA, locally advanced; mAb, monoclonal antibody; NK, natural killer; ORR, overall response rate; OS, overall survival; PCE, paclitaxel, carboplatin, and cetuximab, followed by cetuximab maintenance until PD or toxicity; PD, progressive disease; PD-1, programmed cell death protein 1; PD-L1, programmed cell death ligand 1; PFS, progression-free survival; PI3K, phosphoinositol-3-kinase; R/M, recurrent or metastatic; SCCHN, squamous cell carcinoma of the head and neck; TPE and TPEx, cisplatin, docetaxel, cetuximab.

## References

[1] Siegel R Naishadham D Jemal A. Cancer statistics, 2013. CA Cancer J Clin (2013) 63(1):11–30. 10.3322/caac.21166 23335087

[2] GLOBCAN. GLOBCAN 2012: Estimated Cancer Incidence, Mortality and Prevalence Worldwide in 2012. World Health Organization, International Agency for Research on Cancer Web site (2012). Available from: http://globocan.iarc.fr/Pages/fact_sheets_cancer.aspx

[3] Zhang X Yang H Lee JJ Kim E Lippman SM Khuri FR MicroRNA-related genetic variations as predictors for risk of second primary tumor and/or recurrence in patients with early-stage head and neck cancer. Carcinogenesis (2010) 31(12):2118–23. 10.1093/carcin/bgq177 20819778 PMC3105587

[4] Argiris A Karamouzis MV Raben D Ferris RL. Head and neck cancer. Lancet (2008) 371(9625):1695–709. 10.1016/S0140-6736(08)60728-X 18486742 PMC7720415

[5] Baselga J Trigo JM Bourhis J Tortochaux J Cortés-Funes H Hitt R Phase II multicenter study of the antiepidermal growth factor receptor monoclonal antibody cetuximab in combination with platinum-based chemotherapy in patients with platinum-refractory metastatic and/or recurrent squamous cell carcinoma of the head and neck. J Clin Oncol (2005) 23(24):5568–77. 10.1200/JCO.2005.07.119 16009950

[6] Cho BC Keum KC Shin SJ Choi HJ Lee YJ Kim SH Weekly docetaxel in patients with platinum-refractory metastatic or recurrent squamous cell carcinoma of the head and neck. Cancer Chemother Pharmacol (2009) 65(1):27–32. 10.1007/s00280-009-0999-4 19381630

[7] Price KA Cohen EE. Current treatment options for metastatic head and neck cancer. Curr Treat Options Oncol (2012) 13(1):35–46. 10.1007/s11864-011-0176-y 22252884

[8] Vermorken JB Specenier P. Optimal treatment for recurrent/metastatic head and neck cancer. Ann Oncol (2010) 21(Suppl 7):vii252–61. 10.1093/annonc/mdq453 20943624

[9] Vermorken JB Mesia R Rivera F Remenar E Kawecki A Rottey S Platinum-based chemotherapy plus cetuximab in head and neck cancer. N Engl J Med (2008) 359(11):1116–27. 10.1056/NEJMoa0802656 18784101

[10] Guigay J Fayette J Dillies A-F Sire C Kerger JN Tennevet I Cetuximab, docetaxel, and cisplatin (TPEx) as first-line treatment in patients with recurrent or metastatic (R/M) squamous cell carcinoma of the head and neck (SCCHN): final results of phase II trial GORTEC 2008-03. J Clin Oncol (2012) 30(Suppl):5505.

[11] Tahara M Kiyota N Yokota T Hasegawa Y Muro K Takahashi S Phase II trial of combination treatment with paclitaxel, carboplatin and cetuximab (PCE) as first-line treatment in patients with recurrent and/or metastatic squamous cell carcinoma of the head and neck (CSPOR-HN02). J Clin Oncol (2016) 34(Suppl):6026. 10.1093/annonc/mdy040 29408977

[12] Vermorken JB Trigo J Hitt R Koralewski P Diaz-Rubio E Rolland F Open-label, uncontrolled, multicenter phase II study to evaluate the efficacy and toxicity of cetuximab as a single agent in patients with recurrent and/or metastatic squamous cell carcinoma of the head and neck who failed to respond to platinum-based therapy. J Clin Oncol (2007) 25(16):2171–7. 10.1200/JCO.2006.06.7447 17538161

[13] Mehra R Seiwert TY Mahipal A Weiss J Berger R Eder JP Efficacy and safety of pembrolizumab in recurrent/metastatic head and neck squamous cell carcinoma (R/M HNSCC): pooled analyses after long-term follow-up in KEYNOTE-012. J Clin Oncol (2016) 34(Suppl):6012.

[14] Bauml J Seiwert TY Pfister DG Worden FP Liu SV Gilbert J Preliminary results from KEYNOTE-055: pembrolizumab after platinum and cetuximab failure in head and neck squamous cell carcinoma (HNSCC). J Clin Oncol (2016) 34(Suppl):6011.

[15] Machiels JP Haddad RI Fayette J Afatinib versus methotrexate as second-line treatment in patients with recurrent or metastatic squamous-cell carcinoma of the head and neck progressing on or after platinum-based therapy (LUX-head & neck 1): an open-label, randomised phase 3 trial. Lancet Oncol (2015) 16(5):583–94. 10.1016/S1470-2045(15)70124-5 25892145

[16] Machiels JP Subramanian S Ruzsa A Repassy G Lifirenko I Flygare A Zalutumumab plus best supportive care versus best supportive care alone in patients with recurrent or metastatic squamous-cell carcinoma of the head and neck after failure of platinum-based chemotherapy: an open-label, randomised phase 3 trial. Lancet Oncol (2011) 12(4):333–43. 10.1016/S1470-2045(11)70034-1 21377930

[17] Wainberg ZA Drakaki A. The importance of optimal drug sequencing in metastatic colorectal cancer: biological rationales for the observed survival benefit conferred by first-line treatment with EGFR inhibitors. Expert Opin Biol Ther (2015) 15(8):1205–20. 10.1517/14712598.2015.1050375 26066903

[18] Escudier B Albiges L Sonpavde G. Optimal management of metastatic renal cell carcinoma: current status. Drugs (2013) 73(5):427–38. 10.1007/s40265-013-0043-1 23572408

[19] Bonner JA Harari PM Giralt J Bell D Raben D Liu J PD-036: association of HPV/p16 status with efficacy and safety in pts with OPC in the phase 3 RT/cetuximab registration trial. Radiother Oncol (2014) 114(s1):21 10.1016/S0167-8140(15)34796-4

[20] Taylor RJ Saloura V Jain A Goloubeva O Wong S Kronsberg S Ex vivo antibody-dependent cellular cytotoxicity inducibility predicts efficacy of cetuximab. Cancer Immunol Res (2015) 3(5):567–74. 10.1158/2326-6066.CIR-14-0188 25769300 PMC4681575

[21] Bonner JA Rosenthal DI Mesia R Schulten J Beier F Vermorken JB p16 and human papillomavirus (HPV) subgroup analyses of the IMCL-9815 and EXTREME cetuximab registration trials in squamous cell carcinoma of the head and neck (SCCHN). 7th European Conference on Head and Neck Oncology Budapest (2016).

[22] Agulnik M. New approaches to EGFR inhibition for locally advanced or metastatic squamous cell carcinoma of the head and neck (SCCHN). Med Oncol (2012) 29(4):2481–91. 10.1007/s12032-012-0159-2 22252310 PMC3466428

[23] Trivedi S Srivastava RM Concha-Benavente F Ferrone S Garcia-Bates TM Li J Anti-EGFR targeted monoclonal antibody isotype influences anti-tumor cellular immunity in head and neck cancer patients. Clin Cancer Res (2016) 22(21):5229–37. 10.1158/1078-0432.CCR-15-2971 27217441 PMC5093040

[24] Veluchamy JP Spanholtz J Tordoir M Thijssen VL Heideman DA Verheul HM Combination of NK cells and cetuximab to enhance anti-tumor responses in RAS mutant metastatic colorectal cancer. PLoS One (2016) 11(6):e0157830. 10.1371/journal.pone.0157830 27314237 PMC4912059

[25] Li S Schmitz KR Jeffrey PD Wiltzius JJ Kussie P Ferguson KM. Structural basis for inhibition of the epidermal growth factor receptor by cetuximab. Cancer Cell (2005) 7(4):301–11. 10.1016/j.ccr.2005.03.003 15837620

[26] Goodwin JF Kothari V Drake JM Zhao S Dylgjeri E Dean JL DNA-PKcs-mediated transcriptional regulation drives prostate cancer progression and metastasis. Cancer Cell (2015) 28(1):97–113. 10.1016/j.ccell.2015.06.004 26175416 PMC4531387

[27] Mehra R Cohen RB Burtness BA The role of cetuximab for the treatment of squamous cell carcinoma of the head and neck. Clin Adv Hematol Oncol (2008) 6(10):742–50. 18997665 PMC2745918

[28] Teillaud JL Antibody dependent cellular cytotoxicity (ADCC). eLS (2012). 10.1002/9780470015902.a0000498.pub2

[29] Ferris RL Jaffee EM Ferrone S. Tumor antigen-targeted, monoclonal antibody-based immunotherapy: clinical response, cellular immunity, and immunoescape. J Clin Oncol (2010) 28(28):4390–9. 10.1200/JCO.2009.27.6360 20697078 PMC2954137

[30] Kurai J Chikumi H Hashimoto K Yamaguchi K Yamasaki A Sako T Antibody-dependent cellular cytotoxicity mediated by cetuximab against lung cancer cell lines. Clin Cancer Res (2007) 13(5):1552–61. 10.1158/1078-0432.CCR-06-1726 17332301

[31] Schoppy DW Sunwoo JB. Immunotherapy for head and neck squamous cell carcinoma. Hematol Oncol Clin North Am (2015) 29(6):1033–43. 10.1016/j.hoc.2015.07.009 26568546

[32] Lo Nigro C Monteverde M Etienne-Grimaldi M-C Strola G Lattanzio L Vivenza D Variable impact of chemotherapy ± cetuximab on immune modulation in a prospective cohort of 163 cancer patients. Cancer Res (2015) 75(15 Suppl):1327 10.1158/1538-7445.AM2015-1327

[33] Argiris A EGFR inhibition for recurrent or metastatic HNSCC. Lancet Oncol (2015) 16(5):488–9. 10.1016/S1470-2045(15)70178-6 25892143

[34] Rischin D Spigel DR Adkins D Wein R Arnold S Singhal N PRISM: phase 2 trial with panitumumab monotherapy as second-line treatment in patients with recurrent or metastatic squamous cell carcinoma of the head and neck. Head Neck (2016) 38(Suppl 1):E1756–61. 10.1002/hed.24311 26681429

[35] Vermorken JB Stöhlmacher-Williams J Davidenko I Licitra L Winquist E Villanueva C Cisplatin and fluorouracil with or without panitumumab in patients with recurrent or metastatic squamous-cell carcinoma of the head and neck (SPECTRUM): an open-label phase 3 randomised trial. Lancet Oncol (2013) 14(8):697–710. 10.1016/S1470-2045(13)70181-5 23746666

[36] Apetoh L Ladoire S Coukos G Ghiringhelli F. Combining immunotherapy and anticancer agents: the right path to achieve cancer cure? Ann Oncol (2015) 26(9):1813–23. 10.1093/annonc/mdv209 25922066

[37] Lo Nigro C Ricci V Vivenza D Monteverde M Strola G Lucio F Evaluation of antibody-dependent cell-mediated cytotoxicity activity and cetuximab response in KRAS wild-type metastatic colorectal cancer patients. World J Gastrointest Oncol (2016) 8(2):222–30. 10.4251/wjgo.v8.i2.222 26909137 PMC4753173

[38] Lattanzio L Denaro N Vivenza D Varamo C Strola G Fortunato M Elevated basal antibody-dependent cell-mediated cytotoxicity (ADCC) and high epidermal growth factor receptor (EGFR) expression predict favourable outcome in patients with locally advanced head and neck cancer treated with cetuximab and radiotherapy. Cancer Immunol Immunother (2017). 10.1007/s00262-017-1960-8 28197666 PMC11029535

[39] Baksh K Weber J. Immune checkpoint protein inhibition for cancer: preclinical justification for CTLA-4 and PD-1 blockade and new combinations. Semin Oncol (2015) 42(3):363–77. 10.1053/j.seminoncol.2015.02.015 25965355

[40] Assal A Kaner J Pendurti G Zang X. Emerging targets in cancer immunotherapy: beyond CTLA-4 and PD-1. Immunotherapy (2015) 7(11):1169–86. 10.2217/imt.15.78 26567614 PMC4976877

[41] Heery CR O’Sullivan-Coyne G Madan RA Cordes L Rajan A Rauckhorst M First-in-human phase 1 dose-escalation trial of avelumab. Lancet Oncol (2017). 10.1016/S1470-2045(17)30239-5 PMC6387686 28373007

[42] Seiwert TY Weiss J Baxi SS Ahn M-J Fayette J Gillison ML A phase 3, randomized, open-label study of first-line durvalumab (MEDI4736) ± tremelimumab versus standard of care (SoC; EXTREME regimen) in recurrent/metastatic (R/M) SCCHN: KESTREL. J Clin Oncol (2016) 34(Suppl):TPS6101.

[43] Urba W Chmielowski B Loo D A phase I, open-label, dose escalation study of MGA271 in combination with ipilimumab in patients with B7-H3-expressing melanoma, squamous cell cancer of the head and neck or non-small cell lung cancer. J Immunother Cancer (2015) 3(Suppl 2):176 10.1186/2051-1426-3-S2-P176

[44] Ferris RL Blumenschein GJ Fayette J Guigay J Colevas AD Licitra L Nivolumab for recurrent squamous-cell carcinoma of the head and neck. N Engl J Med (2016) 375(19):1856–67. 10.1056/NEJMoa1602252 27718784 PMC5564292

[45] Chow LQ Haddad R Gupta S Mahipal A Mehra R Tahara M Antitumor activity of pembrolizumab in biomarker-unselected patients with recurrent and/or metastatic head and neck squamous cell carcinoma: results from the phase Ib KEYNOTE-012 expansion cohort. J Clin Oncol (2016) 34(2):3838–45. 10.1200/JCO.2016.68.1478 27646946 PMC6804896

[46] Klochikhin A Greil R Cohen E Vermorken J Harrington K Tahara M Phase 3 trial of pembrolizumab as a first-line treatment in subjects with recurrent/metastatic head and neck squamous cell carcinoma: KEYNOTE-048. Klochikhin (2015) 26(Suppl 8):viii5.

[47] Baughman J Loo D Chen F A phase I, open-label, dose escalation study of MGA271 in combination with pembrolizumab in patients with B7-H3-expressing melanoma, squamous cell cancer of the head and neck, or squamous cell non-small cell lung cancer. J Immunother Cancer (2015) 3(Suppl 2):177 10.1186/2051-1426-3-S2-P177

[48] Rubin Grandis J Melhem MF Gooding WE Day R Holst VA Wagener MM Levels of TGF-alpha and EGFR protein in head and neck squamous cell carcinoma and patient survival. J Natl Cancer Inst (1998) 90(11):824–32. 10.1093/jnci/90.11.824 9625170

[49] Rivera F Garcia-Castano A Vega N Vega-Villegas ME Gutierrez-Sanz L. Cetuximab in metastatic or recurrent head and neck cancer: the EXTREME trial. Expert Rev Anticancer Ther (2009) 9(10):1421–8. 10.1586/era.09.113 19828002

[50] Burtness B Goldwasser MA Flood W Mattar B Forastiere AA Eastern Cooperative Oncology Group. Phase III randomized trial of cisplatin plus placebo compared with cisplatin plus cetuximab in metastatic/recurrent head and neck cancer: an eastern cooperative oncology group study. J Clin Oncol (2005) 23(34):8646–54. 10.1200/JCO.2005.02.4646 16314626

[51] Hitt R Irigoyen A Cortes-Funes H Grau JJ García-Sáenz JA Cruz-Hernandez JJ Phase II study of the combination of cetuximab and weekly paclitaxel in the first-line treatment of patients with recurrent and/or metastatic squamous cell carcinoma of head and neck. Ann Oncol (2012) 23(4):1016–22. 10.1093/annonc/mdr367 21865152

[52] National Comprehensive Cancer Network. Head and Neck Cancers. NCCN Clinical Practice Guidelines in Oncology. Version 2. Fort Washington, PA: National Comprehensive Cancer Network (2016). 10.6004/jnccn.2020.0031 32634781

[53] Guigay J Peyrade F Petre-Lazar B Cetuximab relative dose intensity (RDI) in recurrent/metastatic (R/M) squamous cell carcinoma of the head and neck (SCCHN): first observational prospective study in unselected patients (DIRECT trial). Ann Oncol (2014) 25(s4):iv340–56. 10.1093/annonc/mdu340.11

[54] Gregoire V Lefebvre JL Licitra L Felip E EHNS-ESMO-ESTRO Guidelines Working Group Squamous cell carcinoma of the head and neck: EHNS-ESMO-ESTRO clinical practice guidelines for diagnosis, treatment and follow-up. Ann Oncol (2010) 21(Suppl 5):v184–6. 10.1093/annonc/mdq185 20555077

[55] Knutzen G Subbiah S. Cetuximab rechallenge and monotherapy in patients with squamous cell carcinoma of the head and neck. Case Rep Oncol (2015) 8(3):503–8. 10.1159/000441019 26668572 PMC4677711

[56] Guigay J Fayette J Dillies AF Sire C Kerger JN Tennevet I Cetuximab, docetaxel, and cisplatin as first-line treatment in patients with recurrent or metastatic head and neck squamous cell carcinoma: a multicenter, phase II GORTEC study. Ann Oncol (2015) 26(9):1941–7. 10.1093/annonc/mdv268 26109631

[57] Siano M Resteghini C Cau MC Alfieri S Bergamini C Granata R Outcome of systemic treatments after first line platinum and cetuximab treatment in patients with recurrent/metastatic (RM) head and neck squamous cell cancer (HNSCC): a retrospective analysis. Eur J Cancer (2015) 51:S578 10.1016/S0959-8049(16)31603-3

[58] de Mello RA Geros S Alves MP Moreira F Avezedo I Dinis J. Cetuximab plus platinum-based chemotherapy in head and neck squamous cell carcinoma: a retrospective study in a single comprehensive european cancer institution. PLoS One (2014) 9(2):e86697. 10.1371/journal.pone.0086697 24516537 PMC3916324

[59] Bossi P Kornek G Lanzetta G Rozzi A Füreder T Locati L Safety and feasibility of every-other-week maintenance cetuximab after first-line chemotherapy in patients with recurrent or metastatic head and neck squamous cell cancer. Head Neck (2013) 35(10):1471–4. 10.1002/hed.23170 23042567

[60] Vermorken JB Remenar E Hitt R Kawecki A Rottey S Knierim L Platinum-based chemotherapy (CT) plus cetuximab in recurrent or metastatic squamous cell carcinoma of the head and neck cancer (R/M-SCCHN): 5-year follow-up data for the extreme trial. J Clin Oncol (2014) 32(Suppl):6021.

[61] Peyrade F Cupissol D Geoffrois L Rolland F Borel C Ciais C Systemic treatment and medical management of metastatic squamous cell carcinoma of the head and neck: review of the literature and proposal for management changes. Oral Oncol (2013) 49(6):482–91. 10.1016/j.oraloncology.2013.01.005 23415727

[62] Argiris A Heron DE Smith RP Kim S Gibson MK Lai SY Induction docetaxel, cisplatin, and cetuximab followed by concurrent radiotherapy, cisplatin, and cetuximab and maintenance cetuximab in patients with locally advanced head and neck cancer. J Clin Oncol (2010) 28(36):5294–300. 10.1200/JCO.2010.30.6423 21079141 PMC3018361

[63] Kushwaha VS Gupta S Husain N Khan H Negi MP Jamal N Gefitinib, methotrexate and methotrexate plus 5-fluorouracil as palliative treatment in recurrent head and neck squamous cell carcinoma. Cancer Biol Ther (2015) 16(2):346–51. 10.4161/15384047.2014.961881 25756517 PMC4623054

[64] Argiris A Ghebremichael M Gilbert J Lee JW Sachidanandam K Kolesar JM Phase III randomized, placebo-controlled trial of docetaxel with or without gefitinib in recurrent or metastatic head and neck cancer: an eastern cooperative oncology group trial. J Clin Oncol (2013) 31(11):1405–14. 10.1200/JCO.2012.45.4272 23460714 PMC3612594

[65] Ferris RL Blumenschein GR Fayette J Guigay J Colevas D Licitra LF Further evaluations of nivolumab (nivo) versus investigator’s choice (IC) chemotherapy for recurrent or metastatic (R/M) squamous cell carcinoma of the head and neck (SCCHN): CheckMate 141. J Clin Oncol (2016) 34(Suppl):6009.

[66] Gillison ML Blumenschein GJ Fayette J Guigay J Colevas D Licitra LF Nivolumab (nivo) vs investigator’s choice (IC) for recurrent or metastatic (R/M) head and neck squamous cell carcinoma (HNSCC): CheckMate-141. Cancer Res (2016) 76(14 Suppl):CT099.

[67] Cohen EEW Saba NF Gitlitz B Haddad R Sukari A Neupane P Active8: a randomized, double-blind, placebo-controlled phase 2 study of chemotherapy plus cetuximab in combination with motolimod immunotherapy in patients with recurrent or metastatic squamous cell carcinoma of the head and neck. Presented at: 2016 ESMO Congress; 2016 Oct 7–11; Copenhagen, Denmark [abstract LBA37]. (2016).

[68] Patil VM Noronha V Joshi A Pinninti R Dhumal S Bhattacharjee A Metronomic chemotherapy in platinum-insensitive failures and/or early failures postmultimodality management in oral cancers. Indian J Med Paediatr Oncol (2015) 36(3):161–5. 10.4103/0971-5851.166725 26855524 PMC4743183

[69] Saloura V Cohen EE Licitra L Billan S Dinis J Lisby S An open-label single-arm, phase II trial of zalutumumab, a human monoclonal anti-EGFR antibody, in patients with platinum-refractory squamous cell carcinoma of the head and neck. Cancer Chemother Pharmacol (2014) 73(6):1227–39. 10.1007/s00280-014-2459-z 24714973

[70] Argiris A Buchanan A Brockstein B Kolesar J Ghebremichael M Pins M Docetaxel and irinotecan in recurrent or metastatic head and neck cancer: a phase 2 trial of the eastern cooperative oncology group. Cancer (2009) 115(19):4504–13. 10.1002/cncr.24528 19634157 PMC2749918

[71] Guardiola E Peyrade F Chaigneau L Cupissol D Tchiknavorian X Bompas E Results of a randomised phase II study comparing docetaxel with methotrexate in patients with recurrent head and neck cancer. Eur J Cancer (2004) 40(14):2071–6. 10.1016/j.ejca.2004.05.019 15341981

[72] Vermorken JB Herbst RS Leon X Amellal N Baselga J. Overview of the efficacy of cetuximab in recurrent and/or metastatic squamous cell carcinoma of the head and neck in patients who previously failed platinum-based therapies. Cancer (2008) 112(12):2710–9. 10.1002/cncr.23442 18481809

[73] Herbst RS Arquette M Shin DM Dicke K Vokes EE Azarnia N Phase II multicenter study of the epidermal growth factor receptor antibody cetuximab and cisplatin for recurrent and refractory squamous cell carcinoma of the head and neck. J Clin Oncol (2005) 23(24):5578–87. 10.1200/JCO.2005.07.120 16009949

[74] Jiménez B Trigo JM Pajares BI Sáez MI Quero C Navarro V Efficacy and safety of weekly paclitaxel combined with cetuximab in the treatment of pretreated recurrent/metastatic head and neck cancer patients. Oral Oncol (2013) 49(2):182–5. 10.1016/j.oraloncology.2012.09.003 23026069

[75] Sosa AE Grau JJ Feliz L Pereira V Alcaraz D Muñoz-García C Outcome of patients treated with palliative weekly paclitaxel plus cetuximab in recurrent head and neck cancer after failure of platinum-based therapy. Eur Arch Otorhinolaryngol (2014) 271(2):373–8. 10.1007/s00405-013-2537-6 23644939

[76] Péron J Ceruse P Lavergne E Buiret G Pham BN Chabaud S Paclitaxel and cetuximab combination efficiency after the failure of a platinum-based chemotherapy in recurrent/metastatic head and neck squamous cell carcinoma. Anticancer Drugs (2012) 23(9):996–1001. 10.1097/CAD.0b013e32835507e5 22643048

[77] Knoedler M Gauler TC Gruenwald V Matzdorff A Schroeder M Dietz A Phase II study of cetuximab in combination with docetaxel in patients with recurrent and/or metastatic squamous cell carcinoma of the head and neck after platinum-containing therapy: a multicenter study of the arbeitsgemeinschaft internistische onkologie. Oncology (2013) 84(5):284–9. 10.1159/000345453 23445718

[78] Seiwert TY Fayette J Cupissol D Del Campo JM Clement PM Hitt R A randomized, phase II study of afatinib versus cetuximab in metastatic or recurrent squamous cell carcinoma of the head and neck. Ann Oncol (2014) 25(9):1813–20. 10.1093/annonc/mdu216 24928832 PMC4143093

[79] Cohen EEW Licitra LF Fayette J Gauler TC Clement PM Biomarker analysis in recurrent and/or metastatic head and neck squamous cell carcinoma (R/M HNSCC) patients (pts) treated with second-line afatinib versus methotrexate (MTX): LUX-head & neck 1 (LUX-H&N1). J Clin Oncol (2015) 33(Suppl):6023.

[80] Simpson DR Mell LK Cohen EE. Targeting the PI3K/AKT/mTOR pathway in squamous cell carcinoma of the head and neck. Oral Oncol (2015) 51(4):291–8. 10.1016/j.oraloncology.2014.11.012 25532816

[81] Soulieres D Faivre SJ Mesia R Remenar E Li S-H Karpenko A BERIL-1: a phase II, placebo-controlled study of buparlisib (BKM120) plus paclitaxel in patients with platinum-pretreated recurrent/metastatic head and neck squamous cell carcinoma (HNSCC). J Clin Oncol (2016) 34(Suppl):6008.

[82] Seiwert TY Burtness B Mehra R Weiss J Berger R Eder JP Safety and clinical activity of pembrolizumab for treatment of recurrent or metastatic squamous cell carcinoma of the head and neck (KEYNOTE-012): an open-label, multicentre, phase 1b trial. Lancet Oncol (2016) 17(7):956–65. 10.1016/S1470-2045(16)30066-3 27247226

[83] Delyon J Maio M Lebbe C. The ipilimumab lesson in melanoma: achieving long-term survival. Semin Oncol (2015) 42(3):387–401. 10.1053/j.seminoncol.2015.02.005 25965357

[84] Zandberg DP Strome SE. The role of the PD-L1:PD-1 pathway in squamous cell carcinoma of the head and neck. Oral Oncol (2014) 50(7):627–32. 10.1016/j.oraloncology.2014.04.003 24819861

[85] Zandberg DP Jarkowski A Emeribe UA Goswami T Melillo G A phase 2, multicenter, single-arm, global study of MEDI4736 monotherapy in patients with recurrent or metastatic (R/M) squamous cell carcinoma of the head and neck (SCCHN): HAWK (NCT02207530). J Clin Oncol (2015) 33(Suppl):TPS6086.

[86] Ferris RL Even C Haddad R Tahara M Goswami T Franks A Phase III, randomized, open-label study of durvalumab (MEDI4736) monotherapy, or durvalumab + tremelimumab, versus standard of care (SoC), in recurrent or metastatic (R/M) squamous cell carcinoma of the head and neck (SCCHN): EAGLE. J Immunother Cancer (2015) 3(Suppl 2):150 10.1186/2051-1426-3-S2-P150

[87] Chow LQM Mehra R Haddad RI Mahipal A Weiss J Bergr R Biomarkers and response to pembrolizumab (pembro) in recurrent/metastatic head and neck squamous cell carcinoma (R/M HNSCC). J Clin Oncol (2016) 34(Suppl):6010.

[88] Ratcliffe MJ Sharpe A Rebelatto M A comparative study of PD-L1 diagnostic assays in squamous cell carcinoma of the head and neck (SCCHN). Ann Oncol (2016) 27(Suppl_6):955D 10.1093/annonc/mdw376.07

[89] Champiat S Dercle L Ammari S Hyperprogressive disease (HPD) is a new pattern of progression in cancer patients treated by anti-PD-1/PD-L1. Clin Cancer Res (2016). 10.1158/1078-0432.CCR-16-1741 27827313

